# Improvement of Rotavirus Genotyping Method by Using the Semi-Nested Multiplex-PCR With New Primer Set

**DOI:** 10.3389/fmicb.2019.00647

**Published:** 2019-03-29

**Authors:** Yoshiki Fujii, Yen Hai Doan, Rury Mega Wahyuni, Maria Inge Lusida, Takako Utsumi, Ikuo Shoji, Kazuhiko Katayama

**Affiliations:** ^1^Laboratory of Viral Infection I, Department of Infection Control and Immunology, Kitasato Institute for Life Sciences and Graduate School of Infection Control Sciences, Kitasato University, Tokyo, Japan; ^2^Department of Virology II, National Institute of Infectious Diseases, Tokyo, Japan; ^3^Indonesia-Japan Collaborative Research Center for Emerging and Re-emerging Infectious Diseases, Institute of Tropical Disease, Airlangga University, Surabaya, Indonesia; ^4^Center for Infectious Diseases, Kobe University Graduate School of Medicine, Kobe, Japan

**Keywords:** rotavirus, genotyping, nested-PCR, multiplex-PCR, primer design, equine-like G3

## Abstract

Rotavirus A (RVA) is a major cause of gastroenteritis in infants and young children. After vaccine introduction, RVA surveillance has become more important for monitoring changes in genotype distribution, and the semi-nested multiplex-PCR is a popular method for RVA genotyping. In particular, the VP7 primer set reported by Gouvea and colleagues in 1990 is still widely used worldwide as the recommended WHO primer set in regional and national reference RVA surveillance laboratories. However, this primer set yielded some mistakes with recent epidemic strains. The newly emerged equine-like G3 strains were mistyped as G1, G8 strains were mistyped as G3, the G9 lineage 3 strains showed very weak band, and the G9 lineage 6 strains showed a G9-specific band and a non-specific band. Gouvea’s standard protocol has become relatively unreliable for identifying genotypes correctly. To overcome this limitation, we redesigned the primer set to include recent epidemic strains. Our new primer set enabled us to correctly identify the VP7 genotypes of representative epidemic strains by agarose gel electrophoresis (G1, G2, human typical G3, equine-like G3, G4, G8, G9, and G12). We believe that the multiplex-PCR method with our new primer set is a useful and valuable tool for surveillance of RVA epidemics.

## Introduction

Rotavirus A (RVA), a member of the *Reoviridae* family, is a major cause of gastroenteritis in infants and young children worldwide. In 2016, RVA caused 128,500 deaths in children under 5 years of age globally (Troeger et al., 2018). RVA imposes a huge burden even on developed countries including Japan (Nakagomi et al., 2013). Two live attenuated RVA vaccines, Rotarix (GlaxoSmithKline, Biologicals, Belgium) and RotaTeq (Merck & Co., Inc., United States), were introduced in Japan in November 2011 and July 2012, respectively. The vaccines are very effective (Lambert et al., 2009; Leshem et al., 2014; Karafillakis et al., 2015), but the selective pressure of vaccines may induce an epidemic strain shift.

The RVA genome has 11 gene segments of double-stranded RNA that encode six structural (VPs) and six nonstructural proteins (NSPs) (Estes and Greenberg, 2013). To classify RVAs, a specific genotype is assigned to each of the 11 genome segments, according to predefined nucleotide sequence identity cutoff values (Matthijnssens et al., 2008a,b, 2011). The classification system denotes the VP7-VP4-VP6-VP1-VP2-VP3-NSP1-NSP2-NSP3-NSP4-NSP5/6 genes of an RVA strain as a descriptor Gx-P[x]-Ix-Rx-Cx-Mx-Ax-Nx-Tx-Ex-Hx (x indicating genotype number), respectively. Human RVA strains have been mostly classified into two major and one minor genogroups, based on the genotype constellations (Nakagomi et al., 1989). The Wa, DS-1 and AU-1 genogroups are described as G1-P[8]-I1-R1-C1-M1-A1-N1-T1-E1-H1, G2-P[4]-I2-R2-C2-M2-A2-N2-T2-E2-H2, and G3-P[9]-I3-R3-C3-M3-A3-N3-T3-E3-H3, respectively (Matthijnssens et al., 2008a,b, 2011). Most G1P[8] viruses are thought to possess a Wa-like genotype constellation, and most G2P[4] viruses have a DS-1-like genotype constellation (Heiman et al., 2008).

Since 2012, the unusual DS-1-like G1P[8] strains (G1-P[8]-I2-R2-C2-M2-A2-N2-T2-E2-H2) were detected worldwide, including in Japan (Fujii et al., 2014, 2019; Komoto et al., 2015; Nakagomi et al., 2017; Yamamoto et al., 2017; Jere et al., 2018). This unusual virus has remained one of the common circulating strains in Japan (Sugimoto et al., 2017). In addition, unusual equine-like G3 strains with DS-1-like genotype constellations (G3-P[8]/P[4]-I2-R2-C2-M2-A2-N2-T2-E2-H2) have been detected in many countries, including Australia (Cowley et al., 2016), Hungary (Doro et al., 2016), Spain (Arana et al., 2016), Brazil (Guerra et al., 2016), Thailand (Komoto et al., 2016), and Japan (Malasao et al., 2015). The G8 strains, which were rare in Japan, were also detected at high rates in Hokkaido in 2014 (Kondo et al., 2017). Thus, the genotype distribution in Japan is changing since vaccine introduction.

The semi-nested multiplex-PCR is the popular method for RVA genotyping. Especially, the VP7 primer set reported by Gouvea et al. (1990) is still in use worldwide. In Japan, medical doctors ask the regional institutes of public health for tests of pathogenic agents when needed. The institutes examine specimens and report results to the National Epidemiological Surveillance of Infectious Disease (NESID) system (Taniguchi et al., 2007). For RVA, about half of the reported VP7 genotypes were based on sequencing method, and remaining half were based on the multiplex-PCR and Gouvea’s primer set (calculated from NESID data between 2015 and 2017). However, the primer sequences are hardly updated over the years, and some strains have been reported to be misjudged by this primer set (Mitui et al., 2012). Iturriza-Gomara et al. (2004) improved some of the primer sequences, but the problem of the misjudged cases was not solved, and equine-like G3 and G12 strains cannot be determined by their primer set. Esona et al. (2015) reported a new primer set for VP7 genotyping, but equine-like G3 and G8 strains cannot be determined by their primer set. Nevertheless, the original Gouvea primers are still used in Japan. Therefore, in this study, we showed some misjudged cases and elucidated the mechanisms of errors caused by using that primer set for recent epidemic RVA strains. Then we updated the primer set to make genotyping more accurate.

## Materials and Methods

### Sample Preparation

To evaluate multiplex-PCR methods, we selected 11 representative RVA strains (SP15-09 (Wa-like G1P[8]), NT036 (Wa-like G1P[8]), SP15-06 (DS-1-like G1P[8]), To16-04 (G2P[4]), KN105 (Wa-like G3), To16-01 (equine-like, DS-1-like G3), OH279 (G4P[8]), TA15-07 (G8P[8]), To14-25 (G9 lineage 3), To16-02 (G9 lineage 6), and NS17-5 (G12) that were already genotyped by sequencing (Supplementary Table S1). RNA extraction was performed using the Direct-zol RNA MiniPrep kit (Zymo Research, Irvine, CA, United States) as described (Fujii et al., 2012, 2014).

### Genotyping by Semi-Nested Multiplex-PCR

To avoid false results, we manually designed a new primer set (Table 1) by comparing the VP7 sequences of more than 200 representative RVA strains. Gouvea’s primer set and our new primer set were used for RT-PCR (first PCR) and multiplex-PCR (second PCR). Extracted RNA (1 μL corresponding to 4–106 ng of RNA and 9.5 × 10^-2^–9.0 × 10^-8^ copies of the NSP3 gene) was used for RT-PCR with a TaKaRa One-Step RNA PCR Kit (AMV) (Takara, Kyoto, Japan). Before the reaction, the RNA samples were incubated at 65°C for 5 min with the first primers (10 pmol each). The initial reverse transcription reaction was carried out at 50°C for 30 min and at 94°C for 2 min, followed by 40 cycles of amplification (30 s at 94°C, 30 s at 50°C, 90s at 72°C), with a final extension of 5 min at 72°C in a GenAmp PCR System 2700 thermal cycler (Applied Biosystems, Foster, CA, United States). The first PCR products were diluted 50-fold with DNase/RNase-free water, and the 2 μL of diluted solutions were used for a second PCR. Second PCR was performed using Premix Ex Taq^TM^ Hot Start Version (Takara) with second primers (5 pmol each). The initial denaturation step was at 94°C for 30 s, followed by 20 cycles of amplification (30 s at 94°C, 30 s at 50°C, 60 s at 72°C), with a final extension of 5 min at 72°C. The amplicons were analyzed by electrophoresis on 1.5% agarose gels with ethidium bromide. A 100 bp DNA ladder (New England BioLabs, Ipswich, MA, United States) was used as DNA size marker.

**Table 1:** Primer set for VP7 genotyping.

	Primer	5′- Sequence -3′	position	Product Size (bp)
**Gouvea’s primer set**		
First PCR	Beg 9	GGCTTTAAAAGAGAGAATTTCCGTCTGG	1–28	
	End 9	GGTCACATCATACAATTCTAATCTAAG	1036–1062	1062
Second PCR	RVG 9	GGTCACATCATACAATTCT	1044–1062	
	aAT8 (G8)	GTCACACCATTTGTAAATTCG	178–198	885
	aBT1 (G1)	CAAGTACTCAAATCAATGATGG	314–335	749
	aCT2 (G2)	CAATGATATTAACACATTTTCTGTG	411–435	652
	aDT4 (G4)	CGTTTCTGGTGAGGAGTTG	480–498	583
	aET3 (G3)	CGTTTGAAGAAGTTGCAACAG	689–709	374
	aFT9 (G9)	CTAGATGTAACTACAACTAC	757–776	306
**New primer set (this study)**		
First PCR	VP7 C-040F	CTCCTTTTAATGTATGGTATTGAATATACC	40–69	
	VP7 C-941R	GTATAAAANACTTGCCACCATTTTTTCCA	913–941	902
Second PCR	VP7 C-0932R	ACTTGCCACCATTTTTTCCA	913–932	
	G1-297F	GTATTATCCAACTGAAGCAAGTAC	297–320	636
	G2-401F	TTAAAGACTACAATGATATTACTACATT	401–428	532
	G3-809F	CAAGGGAAAACGTRGCAGTTA	809–829	124
	G3e-757F	CTAGATGTTACTACGGCTAC	757–776	176
	G4-478F	TTCGCTTCTGGTGAGGAGTTG	478–498	455
	G8-179F	TTACRCCATTTGTAAATTCACAG	179–201	754
	G9-606F	GATGGGACARTCTTGTACCATA	606–627	327
	G12-669F	TACRACAACCGACGTCACA	669–687	264


*Some degenerate base symbols have been used (R = A or G, *N* = A, T, G or C)*.

### Sequence Comparison Between Virus Strains and Primers

VP7 nucleotide sequences of representative strains were retrieved from GenBank and aligned using CLUSTAL W, which was included in the MEGA software package, version 7.0.18 and the MAFFT multiple sequence alignment software program, version 7.0 (Katoh et al., 2009). The final edit was performed with Microsoft Excel 2010 software (Microsoft Corporation, WA, United States).

### Ethics Statement

The study protocols were approved by the medical research ethics committee of the National Institute of Infectious Diseases for the use of human subjects. Stool specimens were collected from patients after written informed consent was obtained from the patients or their guardians for the donation of samples.

## Results

### Evaluation of the Gouvea Primer Set

**FIGURE 1:**
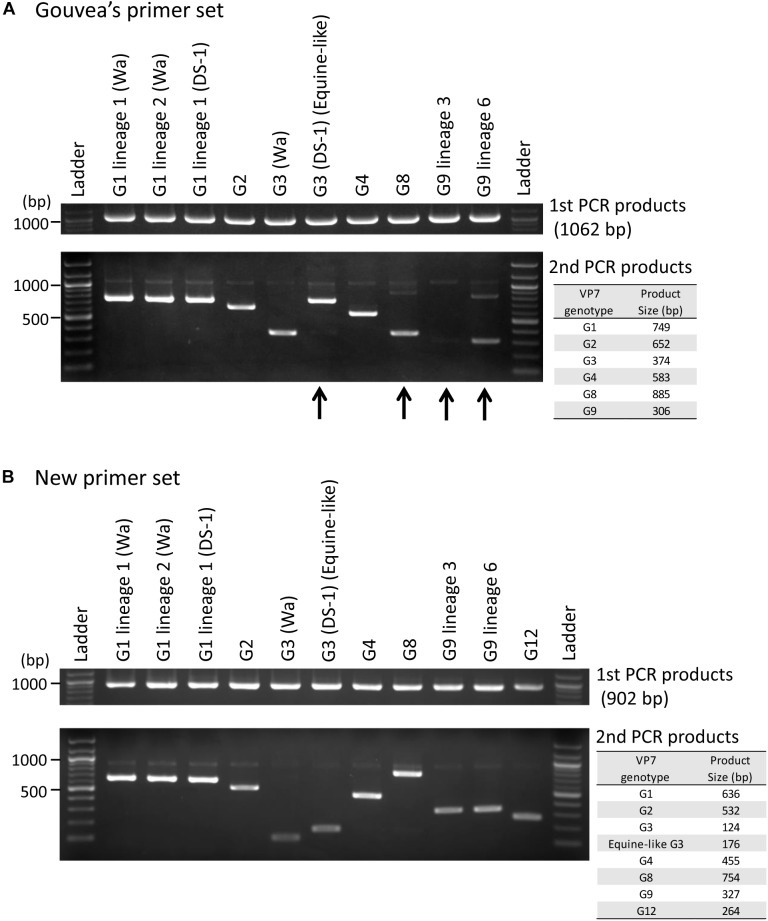
Evaluation of genotyping method using the Gouvea and new primer sets. Japanese representative RVA strains were evaluated by performing RT-PCR (first PCR) and multiplex-PCR (second PCR) with the Gouvea primer set **(A)** or our new primer set **(B)**. The PCR products were analyzed by electrophoresis on 1.5% agarose gels. Estimated product sizes by each primer set are shown in the table to the right. Arrows indicate mistyped cases (equine-like G3 and G8), a difficult to detect case (G9 lineage 3) and a case showing a non-specific band (G9 lineage 6).

Ten representative RVA strains circulating in Japan (Supplementary Table S1, except for G12 strain) were selected for evaluating the Gouvea primer set. Six of 10 strains (G1 lineage 1 (Wa-like), G1 lineage 2 (Wa-like), G1 lineage 1 (DS-1-like), G2, G3 (Wa-like), and G4) were correctly genotyped by their band sizes (Figure 1A). The other four strains (equine-like G3 (DS-1-like), G8, G9 lineage 3 and G9 lineage 6) were mistyped or difficult to identify correctly (Figure 1A). Equine-like G3 strain showed an incorrect band near the size of G1. The G8 strain showed incorrect band at size of G3. The G9 lineage 3 strain showed no band or a very weak specific band that was difficult to identify correctly. In addition, the G9 lineage 6 strain showed the G9-specific band and also a weak non-specific band between sizes of G1 and G8. We confirmed the reproducibility of each failure by using more than five strains (data not shown). To better understand these errors, DNA was purified from each non-specific band and sequenced, and then the mispriming primers were determined (Supplementary Figures S1B, S2B, S3B).

### Evaluation of New Primer Set

Next, using our new primer set (Table 1), we showed that all 11 representative strains (Supplementary Table S1) could be genotyped correctly (Figure 1B). Additionally, we confirmed the reproducibility of these results by using many more strains whose VP7 genes were determined by sequencing (38 G1, 31 G2, 11 human typical G3, 48 equine-like G3, two G4, 14 G8, 32 G9, and three G12 strains, data not shown). The primer for equine-like G3 strains (DS-1-like) is different from that for human typical G3 strains (Wa-like), and these G3 strains can be distinguished by their band sizes (176 and 124 bp).

## Discussion

In this study, we evaluated a semi-nested multiplex-PCR method for VP7 genotyping of RVA with the standard Gouvea primer set and our own new primer set. Of 10 representative Japanese RVA strains, four (equine-like G3 (DS-1-like), G8, G9 lineage 3 and G9 lineage 6) were mistyped or difficult to identify correctly with the Gouvea primers.

To better understand these errors, we compared the primer sequences with VP7 sequences of representative strains of each genotype. As a result, these errors were found to be caused by mispriming of genotype-specific primers (Supplementary Figures S1–S3). The Gouvea G3 primer (aET3) seems to be difficult to bind to the primer binding site (689-709) of equine-like G3 strains because of two substitutions (A705C and A708G) near the 3′ end of this site (Supplementary Figure S1A). The aET3 primer seems to prefer to bind to 292-322 region rather than 689-709 region of equine-like G3 (Supplementary Figure S1B), and induce production of a 771-bp PCR product. We designed G3-809F and G3e-757F primers as new G3 specific primers (Supplementary Figure S1C). Human typical G3 (Wa-like) strains were detected with the G3-809F primer, and equine-like G3 (DS-1-like) strains were detected with G3e-757F primer. These two primers enabled us to distinguish human typical G3 (Wa-like) strains and equine-like G3 (DS-1-like) strains by their band sizes.

For G8 primers, the Gouvea aAT8 primer was based on a 69M strain that is the prototype strain of G8 (Supplementary Figure S2A). However, most epidemic G8 strains have substitutions at the 3′ end of this primer (G198A) and are difficult to amplify with this primer (Supplementary Figure S2B). In addition, as the aET3 primer binds the nucleotides 689–709 of G8 strains, the PCR products of G3 size (374 bp) seem to be preferentially amplified with the Gouvea primer set. The new G8 primer (G8-179F) was designed by shifting three nucleotides to 3′ side and changing mismatched nucleotide (G198A) (Supplementary Figure S2C).

The Gouvea aFT9 primer was based on a WI61 strain (G9 lineage 1) that is the prototype strain of G9 (Supplementary Figure S3A). Most human G9 lineage 6 strains have two substitutions (A759T and C773A), and most G9 lineage 3 strains have three substitutions (A759T, A765G, and C773A). Thus, G9 lineage 3 strains are difficult to amplify with an aFT9 primer. Human G9 lineage 6 strains showed a non-specific band because the aAT8 primer binds the 235–258 region of G9 lineage 6 strains and generates an 825-bp amplicon (Supplementary Figure S3B). As other lineages of G9 strains have a substitution at the 3′ end of the aAT8 primer (G258A), mispriming could occur. The new G9 primer (G9-606F) enables us to detect both of lineage 3 and 6 without non-specific bands (Supplementary Figure S3C).

Our new primer set enables us to correctly identify the VP7 genotypes of most of Japanese epidemic RVA strains, including newly emerged strains (e.g., equine-like G3, G8 and G12). However, RVAs have many genotypes, and inter-species transmission and reassortment sometimes occur (Estes and Greenberg, 2013). Novel RVA strains emerge occasionally. Therefore, the primer sequences should be checked by comparing with the epidemic strains and improved as needed.

Of course, sequencing analysis is important for correct genotyping. However, a great deal of effort, time and money are required for detailed epidemiological surveillance because the genotype distributions can vary based on regions and seasons. The quantitative real-time PCR method (Liu et al., 2015) is not ideal for all institutes because of the costs. Meanwhile, the semi-nested multiplex-PCR method is low-cost and easy to introduce as a routine examination. If many regional institutes of public health can analyze RVA genotypes by multiplex-PCR method and report the results actively, it would be easy to monitor the RVA strains circulating around the country. We believe that the multiplex-PCR method using our new primer set is a useful tool for epidemiological surveillance of RVA.

## Data Availability

The datasets generated for this study can be found in GenBank, LC311226, LC172271, LC311225, LC311229, LC172317, LC311227, LC311231, LC311230, LC105292, LC311228, and LC426752.

## Author Contributions

YF designed new primer set, performed all experiments and was responsible for writing the manuscript. YD, RW, ML, TU, and IS gave assistance to collect samples and analyze RVA sequences. KK gave assistance for the research and helped to draft the manuscript.

## Conflict of Interest Statement

The authors declare that the research was conducted in the absence of any commercial or financial relationships that could be construed as a potential conflict of interest.

## Supplementary Material

FIGURE S1 Sequence comparison of G3 representative strains and primers. Sequences around the Gouvea aET3 primer binding site **(A)**, non-specific binding site of aET3 primer **(B)**, and our new primer binding sites **(C)** are shown. Dots indicate consensus with each primer.

FIGURE S2 Sequence comparison of representative G8 strains and primers. Sequences around the Gouvea aAT8 primer binding site **(A)**, non-specific binding sites of the aET3 primer **(B)**, and our new primer binding site **(C)** are shown. Dots indicate consensus with each primer.

FIGURE S3 Sequence comparison of representative G9 strains and primers. Sequences around the Gouvea aFT9 primer binding site **(A)**, non-specific binding sites of the aAT8 primer **(B)**, and our new primer binding sites **(C)** are shown. Dots indicate consensus with each primer.

TABLE S1 The representative RVA strains used for VP7 genotyping shown in Figure 1.
