# Supplementary figures and images for: Improvement of Rotavirus Genotyping Method by Using the Semi-Nested Multiplex-PCR With New Primer Set
Source: Front Microbiol. 2019 Mar 29;10:647. doi: 10.3389/fmicb.2019.00647 (PMC6449864; doi:10.3389/fmicb.2019.00647)

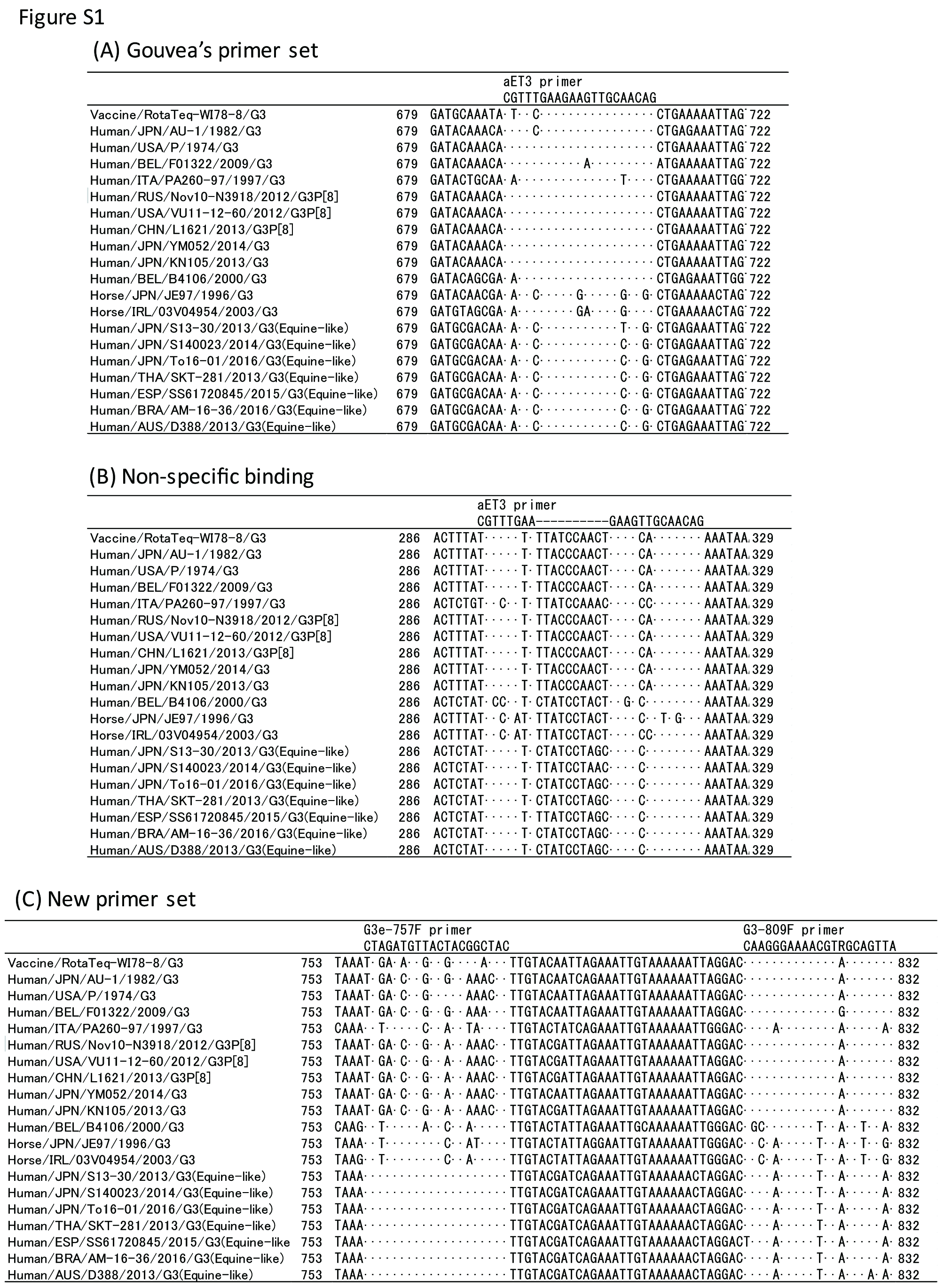

Supplement: FIGURE S1 — Sequence comparison of G3 representative strains and primers. Sequences around the Gouvea aET3 primer binding site (A), non-specific binding site of aET3 primer (B), and our new primer binding sites (C) are shown. Dots indicate consensus with each primer. [file Image_1.TIF]

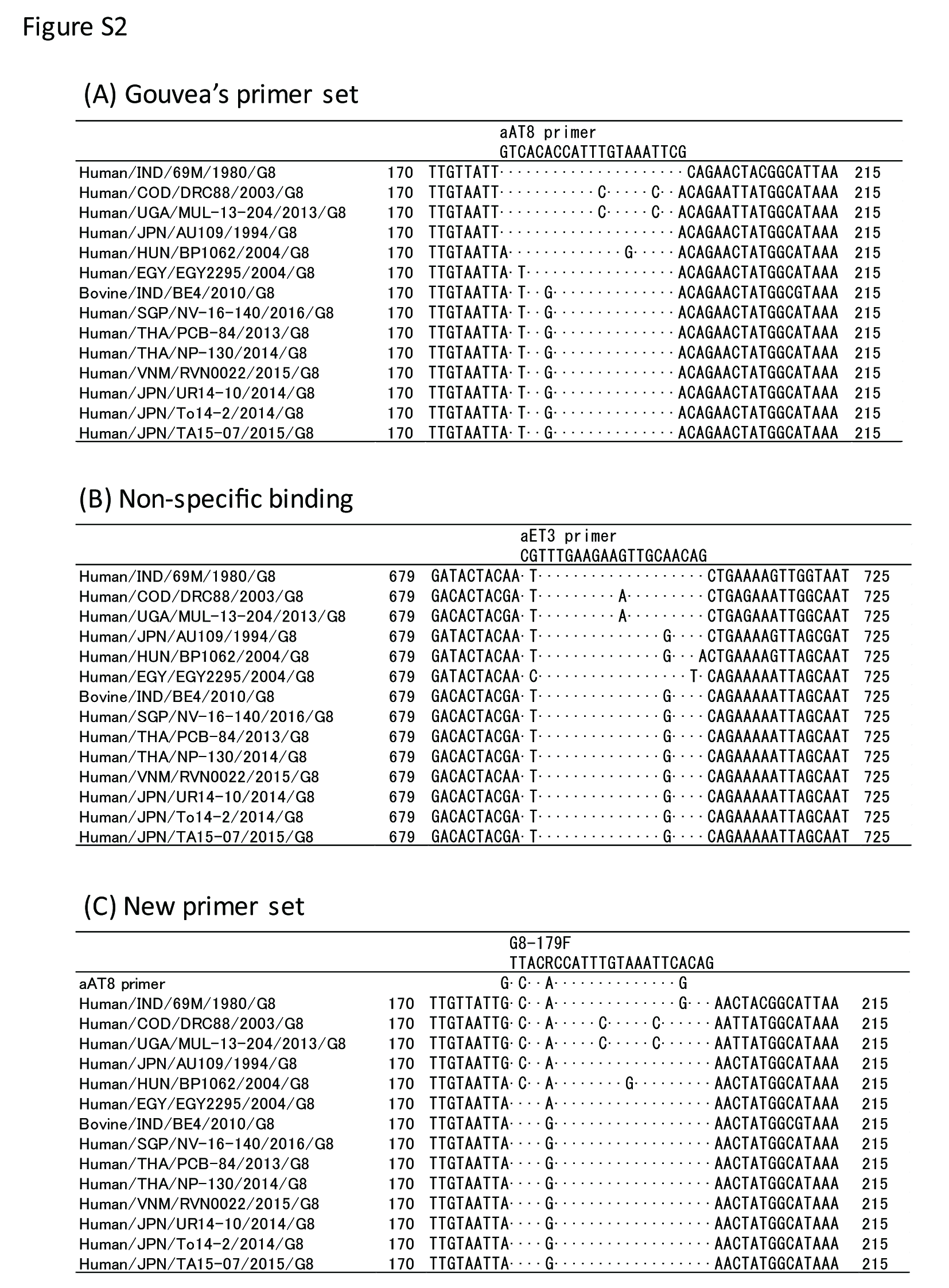

Supplement: FIGURE S2 — Sequence comparison of representative G8 strains and primers. Sequences around the Gouvea aAT8 primer binding site (A), non-specific binding sites of the aET3 primer (B), and our new primer binding site (C) are shown. Dots indicate consensus with each primer. [file Image_2.TIF]

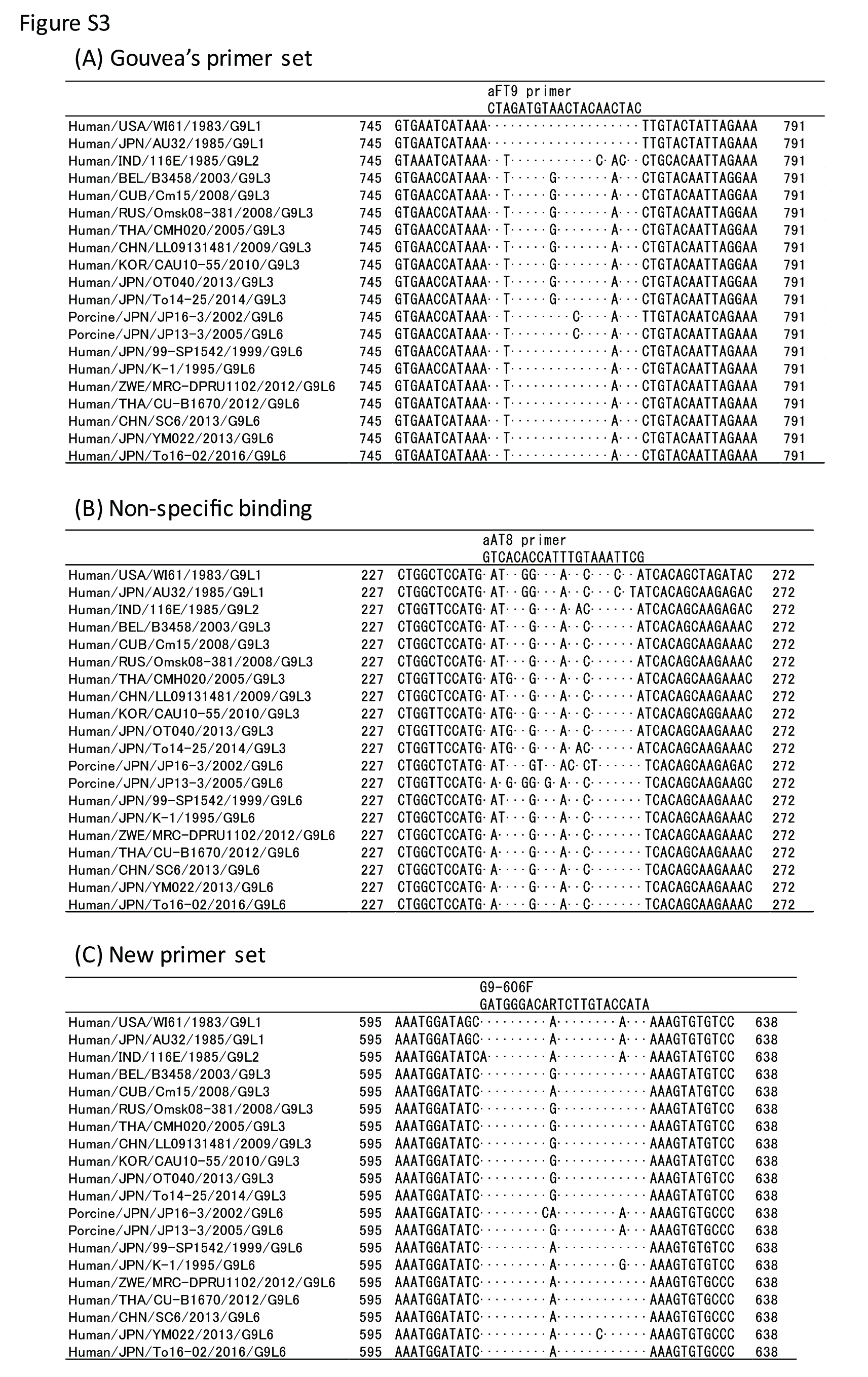

Supplement: FIGURE S3 — Sequence comparison of representative G9 strains and primers. Sequences around the Gouvea aFT9 primer binding site (A), non-specific binding sites of the aAT8 primer (B), and our new primer binding sites (C) are shown. Dots indicate consensus with each primer. [file Image_3.TIF]
