# Supplementary material for: Improvement of Rotavirus Genotyping Method by Using the Semi-Nested Multiplex-PCR With New Primer Set
Source: Front Microbiol. 2019 Mar 29;10:647. doi: 10.3389/fmicb.2019.00647 (PMC6449864; doi:10.3389/fmicb.2019.00647)
Supplement: TABLE S1 — The representative RVA strains used for VP7 genotyping shown in Figure 1. [file Table_1.pdf]

Supplementary Table S1. The representative RVA strains used for VP7 genotyping shown in Figure 1.

Strain name	Genotypes											Remarks	Acc. No. of VP7
	VP7	VP4	VP6	VP1	VP2	VP3	NSP1	NSP2	NSP3	NSP4	NSP5		
RVA/Human-wt/JPN/SP15-09/2015/G1P[8]	G1	P[8]	I1	R1	C1	M1	A1	N1	N1	E1	H1	G1 lineage 1 (Wa-like)	LC311226
RVA/Human-wt/JPN/NT036/2013/G1P[8]	G1	P[8]	I1	R1	C1	M1	A1	N1	N1	E1	H1	G1 lineage 2 (Wa-like)	LC172271
RVA/Human-wt/JPN/SP15-06/2015/G1P[8]	G1	P[8]	I2	R2	C2	M2	A2	N2	T2	E2	H2	G1 lineage 1 (DS-1-like)	LC311225
RVA/Human-wt/JPN/To16-04/2016/G2P[4]	G2	P[4]	I2	R2	C2	M2	A2	N2	T2	E2	H2		LC311229
RVA/Human-wt/JPN/KN105/2013/G3P[8]	G3	P[8]	I1	R1	C1	M1	A1	N1	N1	E1	H1	Human typical G3 (Wa-like)	LC172317
RVA/Human-wt/JPN/To16-01/2016/G3P[8]	G3	P[8]	I2	R2	C2	M2	A2	N2	N2	E2	H2	Equine-like G3 (DS-1-like)	LC311227
RVA/Human-wt/JPN/OH279/2002/G4P[8]	G4	P[8]	I1	R1	C1	M1	A1	N1	N1	E1	H1		LC311231
RVA/Human-wt/JPN/TA15-07/2015/G8P[8]	G8	P[8]	I2	R2	C2	M2	A2	N2	N2	E2	H2		LC311230
RVA/Human-wt/JPN/To14-25/2014/G9P[8]	G9	P[8]	I1	R1	C1	M1	A1	N1	N1	E1	H1	G9 lineage3	LC105292
RVA/Human-wt/JPN/To16-02/2016/G9P[8]	G9	P[8]	I1	R1	C1	M1	A1	N1	N1	E1	H1	G9 lineage6	LC311228
RVA/Human-wt/JPN/NS17-5/2017/G12P[8]	G12	P[8]	I1	R1	C1	M1	A1	N1	T1	E1	H1		LC426752
